# Influence of Strength Level on the Acute Post-Activation Performance Enhancement Following Flywheel and Free Weight Resistance Training

**DOI:** 10.3390/s20247156

**Published:** 2020-12-14

**Authors:** Borja Sañudo, Moisés de Hoyo, G Gregory Haff, Alejandro Muñoz-López

**Affiliations:** 1 Department of Physical Education and Sport, University of Seville, 41013 Seville, Spain; bsancor@us.es (B.S.); dehoyolora@us.es (M.d.H.); 2 Centre for Exercise and Sports Science Research, Edith Cowan University, Joondalup, WA 6065, Australia; g.haff@ecu.edu.au; 3 Departamento de Motricidad Humana y Rendimiento Deportivo, Education Sciences School, University of Seville, 41013 Seville, Spain

**Keywords:** post-activation potentiation, inertial training, vertical jump, sprint test

## Abstract

This study aimed to compare the post-activation potentiation performance enhancement (PAPE) response to the acute inertial flywheel (FW) and free weight resistance training (TRA) on subsequent countermovement jump (CMJ) and sprint performance (10 m sprint). This study used a randomized crossover design including twenty-eight healthy males that were divided into strong (relative one-repetition maximum (1RM) back squat > 2.0 × body mass) and weak (relative 1RM back squat < 2.0 × body mass) groups. All participants performed the following: (a) three reps at 90% of their 1RM back squat (TRA) and (b) three reps on an inertial FW (plus one repetition to initiate flywheel movement) with an intensity that generated a mean propulsive velocity equal to that achieved with 90% of the 1RM back squat. Before and after the conditioning activity, participants performed two CMJs and two 10 m sprints. Within-group analyses showed significantly greater CMJ (*d* > 0.9, *p* < 0.001) and sprint performance (*d* > 0.5, *p* < 0.05) in the FW and the TRA group. Between-group analysis showed that sprint changes were significantly greater in the FW-strong group when compared with the TRA (F_1,18_ = 5.11, *p* = 0.036, η^2^_p_ = 0.221—large) group. These results suggest that using a squat activation protocol on a FW may lead to an acute positive effect on jump and sprint performance, especially in stronger individuals.

## 1. Introduction

An acute enhancement in neuromuscular performance after completing a high-intensity conditioning activity represents what is termed a post-activation performance enhancement (PAPE) [1].Typically, this phenomenon is most noted as improvements in the performance of explosive movements, such as jumping and sprinting [2]. Classically referred to as post-activation potentiation (PAP), this phenomenon can be partially explained by several physiological mechanisms including an (1) increased phosphorylation of myosin light chains, which would render the actin and myosin molecules more sensitive to Ca^2+^ availability [3]; (2) excitation of the central nervous system, leading to increased motor neuron excitability, increased recruitment of high-threshold motor units (fast-twitch fiber contribution), or even increased activation of synergists [4]; and (3) a shortening of the pennation angle, resulting in the improvement of force transfer to the tendon [5].

The improvements in performance after a PAPE inducing stimulus are not consistent within the literature [6], with some studies failing to detect a PAPE response after high-intensity conditioning activities [5,7]. Seitz and Haff [2] suggested that these discrepancies may be partially explained by the structure of the conditioning activity or the athlete’s strength level [5,6,8]. For a conditioning activity that uses traditional weightlifting exercises (i.e., free weights) with high relative loads (i.e., >80% of one-repetition maximum (1RM)), there is a PAPE response as indicated by improvements in subsequent jumping and sprinting performance [5,9,10]. Additionally, fatigue generated in response to the load used during the conditioning activity may be related to the athlete’s relative strength level, which has been reported to modulate the time needed to demonstrate a PAPE response [11]. When athletes do not possess adequate strength levels, long recovery periods (>5 min) between the conditioning activity and the subsequent exercise are needed to maximise the PAPE response [11,12]. Additionally, when shorter rest intervals are placed between the conditioning activity and the performance activity, these athletes may not be able dissipate the fatigue generated by the conditioning activity in order to stimulate a performance enhancement [12]. Based upon this line of reasoning, Seitz and Haff [11] suggest that an athlete’s strength level directly impacts the balance between fatigue and potentiation and dictates how much recovery is required between the conditioning activity and the performance [13]. Therefore, it appears clear that stronger individuals will display a greater PAPE response after shorter recovery periods [11].

Another factor impacting the PAPE response may be the mode of exercise utilised during the conditioning activity [11]. Traditionally, free-weight activities such as squatting [8,9,10] or power cleans [14] have been utilised as part of a conditioning activity that results in an acute PAPE response. Alternatively, a flywheel resistance training device (FRTD) may enhance the amount of work performed during coupled muscle actions [15,16,17]. With these devices, the individual maximally executes the concentric phase against the moment of inertia generated by the system, pulling a rope attached to the rotary shaft [18]. Consequently, the end of the concentric phase is immediately followed by an eccentric phase that results in a greater eccentric muscle activation [19]. This movement pattern results in a very short coupling time (the transition between the concentric and the eccentric phase [20]), which has been reported to be between 0.25 and 0.66 s during the squat exercise when a horizontal cylinder FRTD has been used [16]. This coupling time is different than that seen when traditional free weight squats are performed with a controlled eccentric phase [21]. Some authors use a short pause between both phases (i.e., 2 s) [22] for avoiding the mentioned energy storage, limiting the stretch-shortening cycle (SSC). Consequently, the execution of the exercise can change the stimulation of the SSC, with shortened transition times resulting in greater PAPE [23].

Some recent studies have analyzed the PAPE effects using an FRTD [15,17,24,25,26,27,28,29,30], with several reporting improvements in CMJ [15,24,27], change of direction performance [15,24,28], the standing long jump [24], swimming [25,26], and lower-limb muscle strength [27] performance. For example, Beato et al. [17] reported that using a half squat executed with free weights or a FRTD as a conditioning activity resulted in no differences in performance enhancements during the standing long jump, CMJ, or 5 m sprint time. Conversely, using a similar protocol to Beato et al. [17] (3 × 6 reps at the load that maximized power, with a 3 min rest interval between sets), Timon et al. [30] reported a performance enhancement in the squat jump after performing a conditioning activity that utilized the half squat FRTD and no performance enhancement when a traditional half squat exercise was used as the conditioning activity. While it is difficult to determined why these two studies resulted in different outcomes, the results may be partially explained by differences in the protocols used and the subjects that participated in each study. As such, much more research is needed to determine the impact on the PAPE response when using an FRTD as a conditioning activity.

To the best of our knowledge, no prior study has directly examined the impact of a conditioning activity that uses the squat performed with an FRTD or free-weights on subsequent CMJ and 10 m sprint performance. Additionally, the impact of strength level on the occurrence of PAPE after using an FRTD as a conditioning activity has yet to be examined. Therefore, the primary aim of the present study was to compare the PAPE responses to squats performed with an FRTD or free-weights. Based upon the current body of scientific literature, we hypothesized that the use of an FRTD during a conditioning activity would improve short-term jumping and sprint performance to a greater extent than conditioning activities that utilise free-weights. Our second aim was to determine if the PAPE response induced by an FRTD is related to an individual’s strength level. Based upon the current body of knowledge [11], it was hypothesized that stronger athletes would display a greater PAPE response when compared with weaker athletes.

## 2. Materials and Methods

### 2.1. Participants

Twenty-eight healthy males, with no reported injuries for the past six months (age: 23.5 ± 5.3 years, height: 1.77 ± 0.1 m, mass: 74.3 ± 7.1 kg; 4.0 ± 1.0 years of experience performing lower-limb strength training, absolute 1RM back squat strength: 139.9 ± 27.6 kg), volunteered to participate in the present study. The sample was divided into strong (*n* = 11; age: 23.1 ± 3.5 years, height: 1.76 ± 0.5 m, mass: 70.8 ± 6.6 kg; absolute 1RM back squat strength: 149.0 ± 23.5 kg) and weak (*n* = 17; age: 23.8 ± 6.4 years, height: 1.77 ± 0.6 m, mass: 77.2 ± 5.9 kg; absolute 1RM back squat strength: 112.2 ± 13.1 kg) groups. All participants were recruited from the University Sport Division and via internet advertisements or word of mouth. Participants were recruited on the basis that they engaged in a regular training program and all of them had a training routine of at least three training sessions per week in team sports (i.e., soccer, futsal, and basketball) or individual sports (i.e., athletics) and received normal training during the experiment. Participants with known cardiovascular, metabolic, and/or respiratory disease, or those unable to perform vigorous exercise, were excluded. Further, individuals reporting any lower extremity reconstructive surgeries in the past two years were not included. We informed the participants of the benefits and risks of the investigation, and they voluntarily signed an informed consent document before being involved in this study. We designed and conducted the study following the Declaration of Helsinki and it was approved by a local ethics committee (1044-N-19).

### 2.2. Design

**Figure 1:**
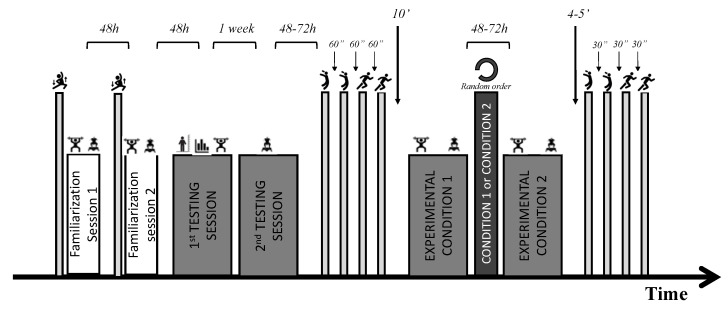
Schematic representation of the study design. 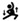: warm up; 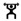: parallel squat exercise; 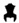: squat with flywheel resistance training device; 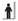: anthropometrics; 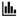: training history; 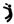: countermovement jump; 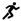: sprint.

We designed a randomized crossover intervention to determine the impact of strength level on the PAPE response during jumping and sprinting performances when a squat using FRTD was compared to a traditional squat protocol using free weights. We divided the participants into two homogeneous groups to compare the same exercises with different resistance training modes. The participants completed two familiarization sessions and were then randomly assigned to four testing sessions. The first experimental condition (free weight) consisted of a parallel squat exercise (TRA—three reps at 90% 1RM), while the second group performed the squat activation protocol on a horizontal-cylinder FRTD (FW—three reps, plus one extra repetition to initiate flywheel movement, with the inertia that generates a Mean Propulsive Velocity (MPV) equal to that reached in the free weight squat at 90% 1RM). In a subsequent session, the order was reversed (Figure 1).

### 2.3. Protocol and Measurements

The study consisted of a two-week testing period. We asked the participants to avoid any strenuous exercise or heavy lower-limb resistance training 24 h before each testing session. Tobacco, alcohol, and caffeine consumption was also prohibited. Further, we instructed all the participants to maintain their regular dietary habits for the duration of the study. All of them participated in six different testing sessions performed at the same time of day and under similar conditions. During the first week, participants visited the laboratory on three different occasions: (a) Monday and Wednesday, participants were provided a familiarization with each testing protocol, including free weights and FRTD equipment. Before any exercise, participants executed a 10 min general warm-up consisting of submaximal running at 9 km·h^−1^, followed by dynamic stretches of the lower-body musculature, half-squat with low loads (two sets of ten repetitions at 50% of body mass), and submaximal familiarization trials with the assessment exercises. During the familiarization visits (sessions 1 and 2), each participant performed each protocol and got familiar with the training equipment and all methodologies used during the investigation. Participants performed five CMJs with progressive intensities and three maximal CMJs, and four 10 m running accelerations at 85%, 90%, 95%, and 100% perceived effort, with 1 min rest periods between them. On Friday (session 3), basic anthropometric measures, including subject age, weight, height, and training history, were collected. Then, the participants performed a progressive resistance loading test for the parallel squat (top of the thigh parallel to the ground). We asked the participants to descend and ascend in a controlled manner without stopping until full extension of the knee and hip joints. An optical encoder was attached to the barbell (ChronoJump Co., Barcelona, Spain; Version 1.7.1.213) with an accuracy ±1 mm and a sampling rate of 1000 Hz to measure displacement during both concentric and eccentric phases. To ensure consistency across attempts and maintain consistency between sessions, we assessed the knee flexion angle using video analysis (Hudl Technique App, Agile Sports Technologies). Participants started from 20 kg in the parallel squat, determining the increments for each load following the protocol outlined by Conceição et al. [31].

A week later (session 4), the participants performed a progressive loading test in the parallel squat using a horizontal-cylinder FRTD (kBox 3, Exxentric, AB TM, Bromma, Sweden), starting with a moment of inertia of 0.025 kg·m^2^. We increased the moment of inertia until 0.45 m·s^−1^, which corresponded to the relative load of 90% of 1RM for the barbell parallel squat. Each repetition consisted of a maximum concentric action accelerating the wheel and, upon completion, decelerating the wheel by means of an eccentric action to stop the movement at about 70° knee flexion [32]. A washout period between 48 and 72 h was allowed. At the same time of day, participants completed the first experimental session (TRA or FW) and, at the next session, the other. The testing days were interspersed with a minimum of 48 h rest in order to limit the effects of fatigue on subsequent tests.

During the fifth and sixth testing sessions, participants executed the experimental conditions. Before the main intervention (conditioning exercise), participants followed a standardized warm-up. Participants were initially seated for 15 min to avoid possible residual fatigue effects and then performed the baseline measures: two CMJs and two 10 m sprint tests prior to each intervention. The rest period between jumps was 60 s, and sprints were interspersed by 60 s recovery periods with another 60 s between exercises. Participants rested for 10 min after the last sprint and then performed the conditioning exercise. Participants allocated to the TRA group performed three repetitions of the barbell parallel squat with a relative load of 90% 1RM. This load was selected based upon previous research that suggests this load stimulates a PAPE response [9,10]. The FW group performed four maximal repetitions (one repetition to initiate the flywheel followed by three maximal repetitions) OF FRTD parallel squat with the individualized moment of inertia previously tested, corresponding to a MPV similar to 90% 1RM for the barbell back squat. We provided verbal encouragement to ensure maximal effort during all repetitions performed. After the exercise, participants rested for 4 min seated on a chair based upon previous literature [33]. Finally, the participants executed the same baseline testing procedures again (Figure 1).

### 2.4. Performance Testing

Vertical jump. We assessed the countermovement jump (CMJ) using a contact mat (ChronoJump, BoscoSystem, Barcelona, Spain) and its proprietary analysis software (ChronoJump 1.7.1.213). All participants performed two CMJ attempts with self-selected countermovement depth. All participants were encouraged to jump as high as possible while placing their hands on their hips in order to remove the contribution of the upper body during the jump. The CMJ technique was emphasized during all testing sessions with the use of demonstrations, verbal cues, as well as a providing familiarization trials during the warm-up. Moreover, all testing was supervised by a certified physical trainer. Sixty seconds of recovery was allowed between each CMJ attempt. We used the highest jump displacement for later analysis. The reliability coefficients of CMJ were fairly high in this study (intraclass correlation coefficient -ICC- = 0.96; 95% confidence interval (CI) = 0.93–0.96; coefficient of variation (CV) = 1.14 ± 0.70%; standard error of measurement (SEM) = 0.24).

Sprint test. All participants performed two maximal 10 m sprint tests on a basketball court measured using dual-beam electronic timing gates (Race Time 2 Light Radio System; Microgate, Bolzano, Italy). The participants were encouraged to sprint as fast as possible, starting at their own volition. The starting position for this test was standardized, with the left toe 0.5 m back from the first gate, which was approximately in line with the athlete’s waist. They performed two trials for each test, with a recovery time of 60 s between attempts. We used the best score for subsequent analysis. The reliability coefficients of the sprint test were fairly high (ICC = 0.92; 95% CI = 0.90–0.94; CV = 2.25 ± 1.97%; SEM = 0.02).

### 2.5. Statistical Analyses

We present the data as mean ± SD. For statistical analyses, participants were divided into *strong* (RM back squat > 2.0 × body mass) and *weak* (relative 1RM back squat < 2.0 × body mass). The 2 times body mass cutoff was selected based on previous studies [11], which have suggested that athletes who have achieved this squat to body mass ratio are able to express a greater degree of PAPE. We performed all statistical power calculations via G*Power 3.1.9.2 (Universität Kiel, Kiel, Germany). Considering an ANOVA test for repeated measures, within-between interaction, the total sample size (*n* = 28), an effect size f of 0.25 (corresponding to a partial eta-squared (η^2^_p_) of 0.06 (medium), an alpha error of 0.05, and 0.9 correlation among repeated measures, we obtained a statistical power of 99%. We evaluated the data normality with the Kolmogorov–Smirnov test. We tested the changes in performance markers about the participant’s strength levels using a two-way mixed repeated-measures analysis of variance (ANOVA) test (time (pre vs. post) and by group (TRA vs. FW) interaction), followed by Bonferroni post hoc test-specific grouping differences. We also calculated the Cohen’s d-effect size (ES, d) statistics and qualitatively analyzed the values following: <0.1, 0.1–0.3, 0.3–0.5, 0.5–0.7, 0.7–0.9, and >0.9, representing trivial, small, moderate, large, very large, and nearly perfect effects, respectively [34]. We also used η^2^_p_ between groups (0.01 = small effect, 0.06 = medium effect, and 0.14 = large effect) to assess the relative magnitude of the differences between groups [34]. Absolute reliability was identified using the standard error of measurement (SEM) and the change in the mean between trials expressed as a coefficient of variation (CV, %) was also determined. Statistical analysis was performed using JAMOVI software (version 0.9, The Jamovi project, 2019) using a significance level of *p* < 0.05.

## 3. Results

The MPV for the parallel squat obtained at 90% 1RM was 0.45 ± 0.02 m·s^−1^, and the inertia eliciting this MPV with the flywheel device was 0.04 ± 0.01 kg·m^2^ (ranging between 0.025 kg·m^2^ and 0.050 kg·m^2^). In Table 1, we show the intra-group changes. Significant changes in the 10 m sprint time were observed both with FW (ES: 0.707, *p* < 0.001) and TRA (ES: 0.466, *p* = 0.025). The CMJ height was also significantly improved in FW (ES: −1.192, *p* < 0.001) and TRA (ES: −0.887, *p* < 0.001) groups. As reported in Table 1, while participants in both FW and TRA groups improved the CMJ, when the participants were divided into *strong* and *weak* groups, only the FW-strong group showed an improvement in sprint time (ES: 1.484, *p* < 0.001).

**Figure 2:**
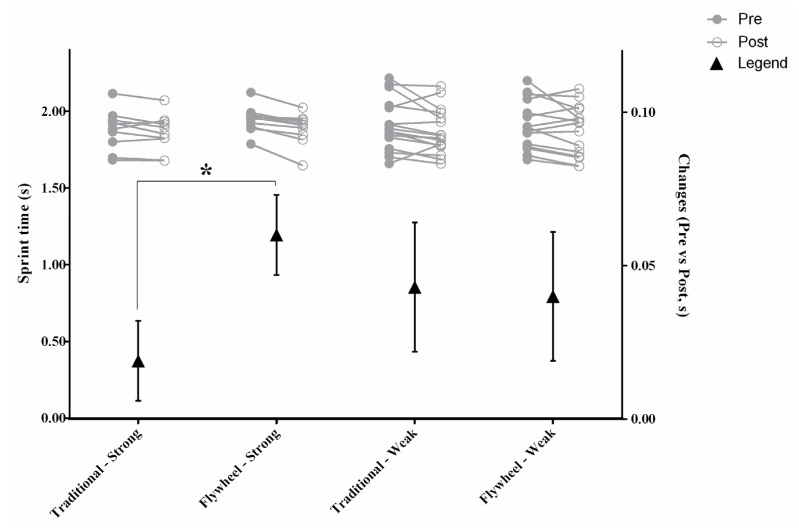
Group condition and individual trend changes from pre to post in sprint. Grey lines represent individual trend changes. Triangle marker represents mean ± standard error group changes. * = significant time*condition interaction (*p* < 0.005).

We also show in Table 1 the between-group changes. There was a group by time interaction (F_1,18_ = 5.11, *p* = 0.036, η^2^_p_ = 0.221—large) in the sprint time, with the post hoc analysis showing a better performance in FW-strong participants when compared with TRA-strong participants (Figure 2).

**Figure 3:**
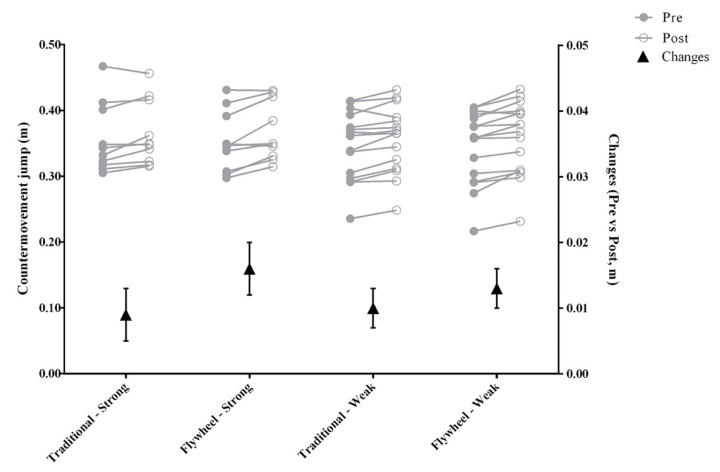
Group condition and individual trend changes from pre to post in countermovement jump (CMJ). Grey lines represent individual trend changes. Triangle marker represents mean ± standard error group changes.

As detailed in Figure 3, there were no significant differences between FW and TRA in CMJ (F_1,50_ = 2.41, p = 0.127, η^2^_p_ = 0.046—small) performance (Figure 3).

## 4. Discussion

The primary purpose of the present study was to determine if there was a difference in the PAPE response when an FRTD or free weight squat is utilised as a conditioning activity. Based upon the present data, there was no difference in improvements in sprint (10 m) or jump (CMJ) performance after either conditioning activity. When strength levels were considered in the present data, stronger participants demonstrate an improved acute sprint performance (10 m) after performing the conditioning exercise with the FRTD. Therefore, we partially confirmed our initial hypothesis that stronger individuals who use the FRTD as a conditioning activity demonstrate an acute PAPE response during subsequent sprinting performance. However, we did not observe a superior PAPE response with this protocol after the jumping performance.

The magnitude of the CMJ improvements in the current study is comparable to previous studies that have reported acute positive effects after performing high-intensity (i.e., 80–95% of 1RM) dynamic resistance exercises [8,10]. These improvements may be attributed to the SSC, typically involved during plyometric exercises, such as jumping [35]. A positive PAPE effect on the jumping performance has been reported when using FRTD [17,24,28,29,30]. Although we observed non-significant differences among protocols in the current study, the positive changes in CMJ (*d* = 1.19—very large effect) performance in response to the FW conditioning exercise were comparable to those reported in the literature using FRTD [36]. Specifically, Maroto-Izquierdo et al. [36] suggested that greater improvements in vertical jump performance were induced after the use of FRTD when compared with traditional resistance training. It seems that, when the recovery time was higher, a superior PAPE effect can be observed in the CMJ using both free weight [37] or FRTD [29]. This lack of significance could also be attributed to the moment of inertia used (i.e., external load or training intensity). In the current study, the training intensity in the FRTD was matched to the barbell squat because it is well documented that, when using free weights, high relative intensities may induce the best acute performance enhancement [2].

The main finding in the current study was that the stronger group displayed a significantly better response in sprint performance (η^2^_p_ = 0.221—large effect) than the TRA group when FRTD was used. Maximum sprint performance is a multifactorial sport-specific task [38] that can be improved with exercises involving the SSC [39]. In our study, we used an FRTD that has been shown to elicit the SSC during the squat exercise [40]. To the best of our knowledge, no previous studies have analysed the acute PAPE effects of this type of activity (i.e., squat using a FRTD device) on sprint performance. Our results showed an improved 10 m sprint performance when using FRTD. Recently, Beato et al. [17] did not found a PAPE response on the 5 m sprinting time when using the same exercise on a similar device, neither using a medium moment of inertia (i.e., 0.029 kg·m^2^) nor a high moment of inertia (i.e., 0.06 kg·m^2^). Because the average moment of inertia we used was similar to them (≈0.04 ± 0.01 kg·m^2^), the differences might be explained by the distance covered (5 m vs. 10 m). In our study, the sprint time over 10 m was improved by 2.1% to 3% in the FW group, while the TRA group displayed a smaller (<2%) improvement. By contrast, Kilduff et al. [37] did not observe increases in 10 m sprint times following a back-squat conditioning activity (3RM = 87% 1RM), which also agrees with Beato et al. [17], who did not find a PAPE effect when using the barbell squat. These differences could again be attributed to the strength levels of the participants. In the current study, we did not observe significant changes in weaker individuals. It has been previously shown that the faster sprinters and stronger athletes have a higher proportion of fast-twitch muscle fibers [41]. In relation to a PAPE response, it can be explained by excitation of the central nervous system, leading to increased motor neuron excitability, more specifically, increased recruitment of the fast-twitch fiber contribution [4].

Previous research showed the positive effects of using an exercise on the vertical plane (i.e., barbell squat) over the performance of a horizontal plane sport-specific task (i.e., sprint) [35]. Suarez-Arrones et al. [42] recently suggested that sprinting, jumping, and squatting performances are different motor qualities to be specifically trained. Finally, the positive PAPE effect found only on the stronger participants when using FRTD can be related to the principle of specificity [43], which suggests that sports performance improves through training movement patterns and intensities of a specific task fitness type [43]. An insufficient stimulation may also explain the difference in the PAPE effects observed in the present investigation in the TRA group. However, the strength level of the participants may have been the main factor dictating the potentiation derived from this type of training. As the conditioning activities require different muscle activation patterns, the PAPE response may be affected by differences in the muscle mechanical work [44]. It seems that the transition between the eccentric and concentric phases during flywheel exercises [45] can induce a greater stretch reflex, where the energy stored during the eccentric phase potentiates the performance of a subsequent concentric action to a greater extent than traditional resistance training exercises. The ability of the FW to produce higher forces at higher velocities [46] may result in an enhanced ability to express high power outputs [16], which has been associated with improved sprint start performance. It is possible that the FW protocol increased activation of the fast-twitch muscle fibers, and thus stimulated a greater PAPE response [19].

Some limitations of our study should be considered. First, recreationally active participants who had been performing lower-body resistance exercise at least twice weekly during the previous two years were recruited for this study. However, we have included strong individuals (i.e., squat RM > 2 times BW). Second, while the rest interval between conditioning and the main activity was controlled in the current study (4 to 5 min rest), small differences in rest between one test and another can affect the PAPE response. Hence, the isolated contribution to jumping or sprint performance requires further study.

## 5. Conclusions

Despite these limitations, the current findings support using a parallel squat using FRTD as a possible conditioning activity for stimulating a PAPE response during CMJ and sprint performances. In addition, the magnitude of improvement is greater in those individuals that were able to squat a minimum of 2 times their body mass. The use of FRTD may be an alternative tool for creating PAPE complexes that target the enhancement of an athlete’s explosive movement capacity.

Strength and conditioning professionals should consider this type of conditioning activity as a potential alternative conditioning activity to the traditional free-weight back squat. Because of the portability of the inertial devices (<15 kg), they may be useful as part of on-field or on-track PAPE complexes in sports, especially in team sports, that require repetitive jumping and sprinting efforts. The results of the current study suggest that the use of a squat protocol using FRTD that includes high-intensity dynamic loading (inertia that generates an MPV equal to that reached in the free weight squat at 90% 1RM) can lead to acute sprint and CMJ performance enhancement. Moreover, stronger individuals demonstrate a greater sprint performance enhancement when compared with PAPE complexes that employ the traditional free weight back squat exercise.

## Figures and Tables

**Table 1:** Sprint and jump performance before and after the two conditioning activities. CMJ, countermovement jump.

		Flywheel				Traditional				Between-Group Comparsions		
		Pre	Post	*p*	*d*	Pre	Post	*p*	*d*	F	*p*	η^2^_p_
CMJ (m)	All	0.35	0.36	<0.001 *	1.192	0.35	0.36	<0.001 *	−0.887	2.410	0.127	0.046
		±0.05	±0.04			±0.05	±0.05					
	Weak	0.35	0.36	<0.001 *	1.218	0.35	0.36	0.002 *	−0.940	0.900	0.350	0.029
		±0.06	±0.06			±0.05	±0.05					
	Strong	0.35	0.37	0.006*	1.135	0.36	0.37	0.038 *	−0.770	1.470	0.241	0.075
		±0.05	±0.04			±0.05	±0.05					
Sprint (s)	All	1.92	1.87	0.001 *	0.707	1.90	1.87	0.025 *	0.466	0.528	0.471	0.010
		±0.14	±0.14			±0.15	±0.14					
	Weak	1.90	1.86	0.063	0.501	1.91	1.87	0.066	0.497	0.007	0.932	0.000
		±0.16	±0.16			±0.17	±0.15					
	Strong	1.95	1.89	0.001 *	1.484	1.88	1.86	0.174	0.467	5.110	0.036 †	0.221
		±0.09	±0.10			±0.13	±0.12					

* Inter-group differences following a *t*-test for dependent measures. † Between-group differences following an analysis of variance (ANOVA) (group x time). *d:* Cohen’s d; η^2^_p_: partial Eta-squared; significant main effects (*p* < 0.05).
